# Elevated PI3K signaling drives multiple Breast Cancer subtypes

**DOI:** 10.18632/oncotarget.285

**Published:** 2011-06-05

**Authors:** Jessica R. Adams, Nathan F. Schachter, Jeff C. Liu, Eldad Zacksenhaus, Sean E. Egan

**Affiliations:** ^1^ Program in Developmental and Stem Cell Biology, The Hospital for Sick Children, 101 College St., East Tower; ^2^ The Department of Molecular Genetics, University of Toronto, Toronto, Ontario, Canada; ^3^ Division of Cell and Molecular Biology, Toronto General Research Institute–University Health Network, Toronto, Ontario, Canada; ^4^ The Department of Medical Biophysics, University of Toronto, Toronto, Ontario, Canada

**Keywords:** PIK3CA, Mouse models, Breast Cancer, PTEN, Akt, Metastasis

## Abstract

Most human breast tumors have mutations that elevate signaling through a key metabolic pathway that is induced by insulin and a number of growth factors. This pathway serves to activate an enzyme known as phosphatidylinositol 3' kinase (PI3K) as well as to regulate proteins that signal in response to lipid products of PI3K. The specific mutations that activate this pathway in breast cancer can occur in genes coding for tyrosine kinase receptors, adaptor proteins linked to PI3K, catalytic and regulatory subunits of PI3K, serine/threonine kinases that function downstream of PI3K, and also phosphatidylinositol phosphatase tumor suppressors that function to antagonize this pathway. While each genetic change results in net elevation of PI3K pathway signaling, and all major breast cancer subtypes show pathway activation, the specific mutation(s) involved in any one tumor may play an important role in defining tumor subtype, prognosis and even sensitivity to therapy. Here, we describe mouse models of breast cancer with elevated PI3K signaling, and how they may be used to guide development of novel therapeutics.

## INTRODUCTION

**Figure 1:**
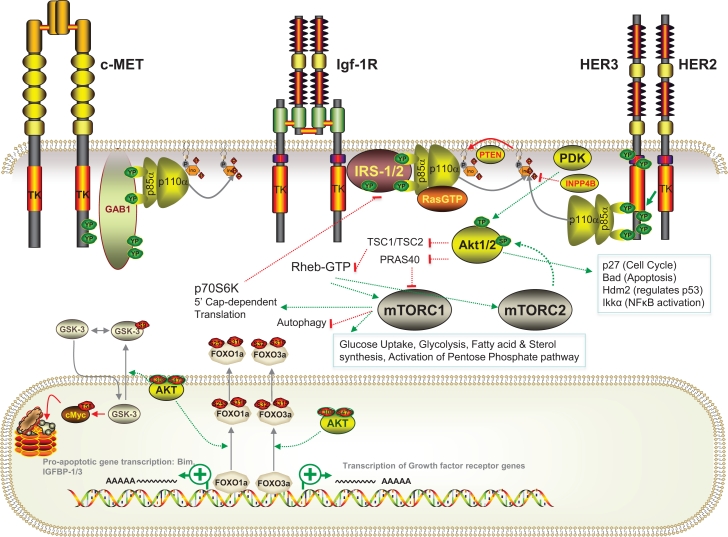
Schematic diagram of the PI3K pathway and its activation in human breast cancer The pathway can be activated at multiple levels including at the level of InsR/Igf-1R, c-MET or HEH2/HER3 receptors, at the level of adaptor proteins, PI3K regulatory or catalytic subunits, at the level of downstream Akt kinases or through deletion/inactivation of inhibitors of the pathway. Note, for simplicity, alternative receptor-activated signaling pathways as well as the full spectrum of PIP3 targets and Akt and TORC targets are not shown.

In 1988, the phosphatidylinositol kinase that copurified with tyrosine kinase receptors was found to phosphorylate phosphatidylinositol lipids at the 3’ hydroxyl position [1, 2]. This enzyme, class I phosphatidylinositol 3’ kinase (PI3K), was subsequently found to be responsible for converting phosphatidylinositol 4,5-bisphosphate (PIP2) to phosphatidylinositol 3,4,5-trisphosphate (PIP3), and has been implicated in biological processes from insulin-mediated regulation of glucose uptake and metabolism to transformation and even metastatic dissemination of tumor cells [1]. Class IA PI3Ks are composed of one regulatory and one catalytic subunit. The most frequently expressed and commonly studied regulatory subunit is p85α. This binds to the p110α catalytic subunit to control its stability and activity [3]. PI3K signaling is stimulated in response to activation of many growth factor receptors, most potently by the insulin receptor tyrosine kinase (InsR) or related insulin-like growth factor 1 receptor (IGF-1R) (Figure 1). In either case, receptor activation leads to tyrosine phosphorylation of a large adaptor protein from the insulin receptor substrate family (IRS1, 2, 3 or 4) [4]. The IRS proteins contain a number of YxxM motifs that, when phosphorylated on tyrosine (Y), form high affinity binding sites for certain SH2 domains such as those found within the p85 regulatory subunit [5]. Recruitment of the p85;p110 PI3K complex to tyrosine-phosphorylated IRS overrides the inhibitor influence of p85 on its catalytic partner [3, 5, 6]. Binding of GTP-loaded Ras to p110 also increases kinase activity [7]. These effects synergize, and the resulting activated PI3K converts PIP2 into PIP3 [7, 8]. PIP3 subsequently recruits, and in some cases activates, a series of signaling proteins, most of which contain PIP3-binding pleckstrin homology (PH) domains. Best studied among these PIP3 targets are the Akt (1, 2 3)/PKB (α, β or γ) AGC-family serine/threonine kinases [9-11], as well as PDK1 which phosphorylates Akt at threonine 308 (Akt1), thereby activating it with respect to a number of substrates including PRAS40 and TSC2. Phosphorylation of PRAS40 by Akt induces sequestration of p-PRAS40 by 14-3-3 proteins, which prevents it from inhibiting the mTOR, Raptor, mLST8 and Deptor-containing TORC1 serine/threonine kinase complex [12-14]. Similarly, Akt-mediated phosphorylation of TSC2 leads to suppression of the TSC1/TSC2 Rheb GAP activity with subsequent accumulation of GTP on the Rheb small GTPase [15]. Rheb-GTP activates TORC1. Thus, phosphorylation of PRAS40 and TSC2 lead to activation of TORC1, which blocks autophagy while increasing cap-dependent protein translation, glucose uptake, glycolysis, activation of the pentose phosphate pathway as well as fatty acid and sterol synthesis [16-20]. TORC1 is also regulated by Rag-family GTPases that respond to amino acid levels, AMP kinase that is regulated by the AMP:ATP ratio as a readout of cellular energy levels, as well as by the Rac GTPase that functions downstream of growth factor signaling [21]. Full activation of Akt also requires phosphorylation at serine 473 by TORC2, a TORC1-related complex containing mTOR, Rictor, mLST8, Deptor, mSIN and Proctor [22]. TORC2, or a related Rictor-containing complex, also contains integrin linked kinase (ILK) [23-26]. ILK is a kinase/adaptor protein that binds to β1-integrins as well as to PIP3, and is required for recruitment of caveolae to the plasma membrane [23, 25, 27, 28]. Akt^pT308/pS473^ phosphorylates many signaling proteins, including GSK3 serine/threonine kinases and Hdm2 E3 ubiquitin ligases, inhibiting the former and activating the latter [10, 29]. Akt also phosphorylates FOXO1a/3a transcription factors, which causes FOXO-14-3-3 complex formation and nuclear exclusion, thereby blocking the ability of FOXO proteins to activate a pro-apoptotic transcriptional program [22, 30]. Indeed, Akt regulates survival on the level of transcription, through FOXO, and also through phosphorylation of cytoplasmic proteins including Bad [10].

Both constitutive and inducible negative regulators act to antagonize the PI3K pathway, maintaining the system in an off state under resting conditions and returning it to this state once stimulation is relaxed (Figure 1). These include ligand-sequestering proteins as well as tyrosine phosphatases and endocytic regulators that function to shut down receptor signaling. In addition, PIP3 levels are held in check by phosphatases that remove the 3’ phosphate, the 4’ phosphate or the 5’ phosphate. The most direct negative regulator of this pathway is PTEN, a phosphatidylinositol 3’ phosphatase and limited specificity protein phosphatase [31-35]. PTEN was discovered as a tumor suppressor that, when deleted, leads to elevated levels of PIP3. Interestingly, PTEN also functions in the nucleus as a cell cycle inhibitor via its ability to positively regulate the anaphase promoting complex (APC-CDH1) [36, 37]. Of note, type II phosphatidylinositol 4’ phosphatase also functions to limit signaling though PI3K pathway targets and is deleted in some tumors (see below) [38]. Other inhibitors of this pathway include PHLDA3, a p53-inducible PH domain protein that sequesters PIP3 and blocks activation of Akt [39], as well as the PH-domain containing PHLPP serine/threonine phosphatases that dephosphorylate Akt at serine 473 [40]. In addition to PRAS40 and TSC2, which limit TORC1 activation as noted above, the Dep and PDZ domain protein, Deptor, is a potent inhibitor of TORC1 and TORC2 [40, 41]. Interestingly, Deptor can enhance the TORC2/TORC1 activity ratio in cells [41]. Finally, several PI3K pathway kinases function as feedback inhibitors to shut down signaling. For example, activated Akt can inhibit FOXO-mediated expression of growth factor receptors including InsR, IGF-1R and HER3 [30, 42]. The TORC1 target, p70S6 Kinase (S6K1) can phosphorylate and target IRS proteins for degradation, and thereby choke off further insulin-mediated activation of PI3K [4, 43].

## THE PI3K PATHWAY IS ONCOGENIC

The PI3K pathway has been linked to growth control and transformation in many tissues [19]. Indeed, genes coding for most of the proteins described above are oncogenes or tumor suppressor genes, depending on whether they function to activate signaling through the PI3K pathway or act to inhibit it. The gene coding for p110α, *PIK3CA*, was identified as a viral oncogene in Avian Sarcoma Virus 16 [44, 45]. This discovery presaged identification of *PIK3CA* mutations in many human tumors, most prominently in tumors of the breast, colon, endometrium and thyroid gland [11, 45-47]. Mutations in this gene map to two hotspots, one coding for a centrally located helical domain, typically E542K or E545K, and one in the C-terminal kinase domain, most commonly H1047R [46]. Surprisingly, while helical domain and kinase domain mutations both increase the catalytic activity of p110α, they show differing requirements for full activation *in vivo*. Specifically, helical domain mutants still depend on Ras-GTP for activation, but not on p85. In contrast, full activation of the H1047R kinase domain mutant is Ras-independent, but dependent on p85-phosphotyrosine interaction [48]. Indeed, helical and kinase domain mutations cooperate when present in the same cDNA, generating an allele capable of activating Akt/PKB to a level 1000-fold higher than observed downstream of either single mutant [48]. These mutations are not commonly seen together in the same tumor. However, *PIK3CA* mutations do occur with mutations that activate tyrosine kinases, activate Ras or inactivate PTEN [49]. Thus, greatly enhanced PI3K signaling may only be achieved through cooperating oncogenic mutations that override negative regulation of this biologically powerful pathway. In this regard, p53 and the PI3K pathway intersect at multiple levels. For example, Akt activates the Hdm2 E3 ligase that targets p53 for destruction [50, 51], and conversely, p53 induces expression of PI3K pathway inhibitors, PTEN and PHLDA3 [39, 52]. These interactions may well explain the coincidence of mutations that affect both pathways in many tumors (see below).

## MUTATIONS IN PI3K PATHWAY IN HUMAN BREAST CANCER

With the advent of tumor re-sequencing, commonly mutated oncogenes, tumor suppressor genes and defective signaling pathways involved in many tumor types have been identified. This analysis yielded a somewhat disappointingly complex picture for breast cancer, where a large number of mutations have been identified, each in a small percentage of tumors [53]. There were, however, two genes mutated in a large fraction of breast tumors: *TP53* and *PIK3CA* [53]. For example, mutant alleles of *PIK3CA* were identified in approximately 30% of breast tumors [46, 54-56]. The specific alleles found include both helical and kinase domain mutants, each occurring with approximately the same frequency. In some cases, *PIK3CA* gene amplification was also noted [57]. This was more common in tumors with helical domain mutant alleles [58].

The PI3K pathway is also activated in breast cancer through copy number changes and/or mutations or deletions in several other genes [59-63] (Figure 1). For example, the gene coding for HER2/Neu is amplified and frequently associated with high-level expression of HER3, a pseudokinase receptor, HER2-binding partner and substrate with multiple YxxM sites for recruitment of p85 [64]. Some breast carcinomas show gene amplification at the *MET*/*CAVEOLIN* gene locus [65-68]. This results in elevated tyrosine kinase signaling from MET through to Gab adaptor proteins [69, 70] and potentially to HER3 [71], both of which bind p85 when phosphorylated [64, 69, 70]. Caveolin proteins enhance InsR and IGF-1R signaling [72-74]. Indeed, the *InsR* and *IGF-1R* genes are amplified in some breast tumors [75, 76]. Genes coding for cytoplasmic adaptor proteins like IRS-4 as well as Gab1 and Gab2 are mutated or amplified in a small percentage of breast cancers [53, 77-79]. *PIK3R1*, the gene coding for p85α, is also mutated in some cases [53, 78]. Downstream of *PIK3CA*, gene amplifications occur in *PDPK1*, the gene coding for PDK1 [80]. Also, gain-of-function, activating mutations were found in the PH domain of *AKT1 (AKT1^E17K^)* [81, 82].

Mutations that disrupt negative regulators of the PI3K pathway have also been detected in breast cancer. For example, the gene coding for PTPN12/PTP-Pest, a non-receptor tyrosine phosphatase, is commonly disrupted, leading to enhanced tyrosine phosphorylation of multiple growth factor receptors, with resulting downstream PI3K pathway activation [83]. Heterozygous loss-of-function germline mutations in *PTEN* cause PTEN hamartoma tumor syndromes (PHTS) including Cowden’s syndrome that is associated with a high incidence of breast cancer [84]. Accordingly, approximately 30% of sporadic breast tumors show *PTEN* inactivation, either through mutation or epigenetic suppression [31, 32, 84-88]. miR-21 has been shown to suppress PTEN gene expression in response to IL6/Stat3 signaling in many breast tumors [89-92]. HER2-mediated activation of the Src tyrosine kinase causes phosphorylation of PTEN and its dissociation from the plasma membrane, thereby enhancing PI3K pathway signaling [93, 94]. Finally, as noted above, loss-of-function mutations in type II phosphatidylinositol 4’ phosphatase (*INPP4B*) also occur in breast cancer [38, 95]. As more breast cancer genomes are sequenced, it is not unreasonable to expect evidence for each and every PI3K pathway regulatory gene to be implicated in a subset of breast tumors.

## THE PI3K PATHWAY AND BREAST CANCER SUBTYPES

The diagnosis of breast cancer describes a collection of diseases. The distinction between hormone receptor positive and negative forms dates back many years, and pathologists have long noted a wide range of histological and clinical features in breast cancer. However, with advances in transcriptional profiling, a relationship between pathological subtype and what is now called molecular subtype has emerged [96]. Equally exciting is the realization that less common mutations in breast cancer as a whole can be quite common when individual breast cancer subtypes are considered [97]. The major molecular subtypes are: luminal A and B, HER2^+^, basal and claudin-low. Luminal A and B are both estrogen receptor (ERα) positive subtypes, whereas basal and claudin-low are triple negative tumors (ERα-negative, progesterone receptor negative and HER2 negative) [98].

*PIK3CA* mutations are found in tumors from most subtypes, which explains why this gene scored as one of the two most commonly mutated genes in breast cancer [53-56]. For example, 35% of estrogen receptor (ERα) positive tumors, 23% of HER2/Neu positive tumors and 8% of basal tumors have *PIK3CA* mutations [99]. *PIK3CA* mutations either do not occur, or occur at a very low frequency, in claudin-low breast cancer [99]. In addition to the major subtypes, there are a number of rare pathological variants of breast cancer that are not represented in most studies. Indeed, metaplastic breast cancer, a relatively rare form [100], shows the highest frequency of *PIK3CA* mutations (47%) [99]. Finally, *PIK3CA* mutations are found in many papillary breast tumors and in androgen receptor positive apocrine breast tumors, as well as in premalignant lesions such as DCIS [101-104]. Remarkably, breast cancers with helical and kinase domain mutant alleles show widely differing prognoses. Helical domain mutations are associated with dramatically reduced overall and disease-free survival, whereas patients with kinase domain mutant breast tumors show enhanced survival as compared to patients with either wildtype or helical mutant *PIK3CA* [105]. In line with this finding, expression of an E545K helical domain mutant of *PIK3CA* in MDA-MB-231 cells induced a highly motile and malignant state, in contrast to the effect of expressing an H1047R allele, which caused more limited transformation [106].

With additional transcriptional profiling, the 5 molecular subtypes have been further subdivided on the basis of signaling pathway activation to 17 identifiable groups of tumors [97]. This analysis has shown that PI3K pathway activity is elevated in over half of the luminal subtypes, in 1 of 2 HER2/Neu subtypes and 3 of 3 basal subtypes. Particularly striking is the very high level of PI3K pathway activation observed in luminal B subtype 6, suggesting that mutations in several genes may cooperate to hyperactivate the pathway in these tumors [97].

In contrast to *PIK3CA* mutations, some PI3K pathway mutations are found in a limited group of breast tumors because they are associated with one or few subtypes. For example, and by definition, high-level expression of HER2/Neu activates PI3K signaling in HER2^+^ subtype tumors. Loss of *PTEN* gene function or expression is frequently observed in basal-like breast tumors [107, 108]. In addition, *INPP4B* is preferentially lost in basal breast cancers [95, 109]. In contrast, activating mutations in Akt1 are most frequently observed in luminal tumors and specifically in papillary tumors [101].

Some forms of breast cancer show very high level PI3K pathway activation [97], and this situation is associated with poor survival [61, 62]. Initially, it was thought that different mutations in the pathway would be mutually exclusive and unnecessary to achieve transformation. However, this idea has proved to be incorrect and cooperation between several oncogenic mutations on the pathway is relatively common, especially in poor prognosis tumors [49]. For example, amplification of *HER2* and mutational activation of *PIK3CA* or *PTEN* inactivation occur together in many breast tumors [56]. This situation has been associated with resistance to HER2 targeted therapy [93, 94, 110-112]. Also, in MCF10A cells expressing high levels of HER2/Neu, a kinase domain mutant of *PIK3CA* (H1047R) induced expression of Heregulin, the ligand that activates HER2/HER3 signaling through the PI3K pathway [113]. In contrast, a helical domain mutant (E545K) enhanced transformation without inducing Heregulin expression [113]. Therefore, in HER2 subtype breast cancers with *PIK3CA^H1047R^* or other kinase domain mutant alleles, therapy with Herceptin/Trastuzumab together with an antibody that blocks Heregulin could be particularly effective. As noted above, the gene coding for PDK1 is amplified in many breast tumors with PI3K pathway activation, including tumors with HER2 amplification, activating mutations in *PIK3CA* or *PTEN* inactivation [80]. *PTEN* inactivation and *PIK3CA* mutation occur together in a subset of tumors [1], as do *PTEN* inactivation and inositol polyphosphate 4-phosphatase II gene deletion [109].

## MOUSE MODELS

Mouse models of breast cancer have been refined through use of gene targeting to generate conditional mutants and transgenics that mimic pathological features of specific breast cancer subtypes [114-118]. To model *PIK3CA*-mutant breast cancer we generated mice with an H1047R mutant *Pik3ca* cDNA targeted to the ubiquitously expressed ROSA26 locus (R26) [119]. This cDNA was preceded by 5’ loxP-flanked transcriptional stop sequences and, when mated to MMTV-Cre mice, approximately 70% of the resulting female R26-Pik3ca^H1047R^;MMTV-Cre mice developed mammary adenosquamous carcinoma or adenomyoepithelioma starting at about 5 months of age. Control R26-Pik3ca^wt^;MMTV-Cre females were also generated but these animals did not develop mammary tumors. Glandular regions of the H1047R mutant tumors included cells expressing luminal and basal epithelial markers, whereas squamous regions expressed mesenchymal markers such as vimentin, desmin and/or N-cadherin. A subset of glandular cells expressed the estrogen receptor, which matches molecular subtype data in humans, where *PIK3CA* mutations are commonly found in ERα-positive luminal breast cancers. As expected, tumors from this mouse model showed evidence of PI3K/Akt pathway activation. To test for cooperation between *PIK3CA* and *TP53*, the two most common mutations in breast cancer [53], R26-Pik3ca^H1047R^;MMTV-Cre mice were also crossed with p53^loxP^ conditional mutants [120]. The resulting double mutant females showed accelerated tumor onset as well as an altered spectrum of mammary tumors [119]. A second model of *PIK3CA^H1047R^*-induced breast cancer has also been reported. In this study, a ROSA-targeted H1047R mutant cDNA was activated by expression of either WAP-Cre or MMTV-Cre. Mammary tumors in this model, which were ERα-positive and contained cells expressing either cytokeratin type as above, also showed evidence of enhanced PI3K/Akt pathway signaling. These mice developed mammary adenosquamous carcinomas and adenomyoepitheliomas, as well as adenocarcinomas with squamous metaplasia, adenocarcinomas and adenocarcinmatosis with invasive periductal cords of neoplastic cells [121]. Thus, in both cases, Pik3ca^H1047R^ induced a heterogeneous mixture of ERα-positive mammary tumors, some of which showed metasplastic differentiation. These data fit with the wide spectrum of *PIK3CA* mutant breast tumors observed in humans. However, these results contrast studies with activated Akt. Several groups have generated transgenic mice expressing mutationally activated Akt1 in the mammary gland. In each case, mutant Akt1 delayed involution but did not induce tumor formation [122, 123]. As with R26-Pik3ca^H1047R^-Cre model mice, but unlike Akt1 transgenics, *Pten* loss-of-function mutants develop mammary tumors. This was first observed in *Pten* heterozygous mice that model Cowden’s syndrome [124], but also confirmed in Pten^loxP^;MMTV-Cre conditional mutants which developed mammary tumors starting at 2 months of age [125]. These tumors ranged from benign fibroadenomas to pleiomorphic adenocarcinomas [125]. Finally, mammary tumors were also induced in transgenic mice overexpressing Igf1R in mammary epithelium [126]. A heterogeneous mixture of tumor types was also observed in this model, with adenosquamous carcinoma and adenomyoepithelioma occurring at a high frequency. In addition, more homogenous HER2/Neu-like tumors were also noted as were metaplastic Wnt-like tumors [126, 127].

Mouse studies have also revealed cooperative interaction between PI3K pathway genes and other genes or pathways implicated in human breast cancer. As noted above, *TP53* deletion showed cooperative interactions with *Pik3ca^H1047R^* [119]. Also, a dominant *Akt1* mutant reduced latency of tumor formation in MMTV-Neu mice, while decreasing invasion and metastasis in this model [128]. In contrast, deletion of *Pten* decreased tumor latency in MMTV-Neu mice but induced development of heterogeneous basal-like mammary tumors with enhanced metastatic dissemination [129]. These results were somewhat surprising and revealed further complexity in PI3K pathway signaling. Indeed, *in vitro* studies have shown that, like p110α [130], Akt2 activation is associated with enhanced β1-integrin mediated migration [131]. In fact Akt1 and Akt2 appear to play opposite roles in this context, with Akt1 suppressing Akt2-dependent migration [132]. *In vivo*, Akt1 enhanced growth of HER2/Neu primary tumors, but suppresses their dissemination, whereas Akt2 impairs local growth but stimulated metastasis [133-135]. The mechanism by which Akt isoforms regulate migration and metastasis in opposite directions may involve differential regulation of Pak kinase by Akt1 and Akt2 and/or distinct subcellular localization [134, 136, 137]. Also, Akt1 signaling maintains high expression of miR-200-family microRNAs that suppress epithelial mesenchymal transition and Akt2 upregulates miR-21, which inhibits PTEN expression as noted above [138, 139]. On the other hand, differential activation of Akt1 and 2 may be achieved through PHLPP serine/threonine phosphatases. PHLPP1 dephosphorylates and thereby inactivates Akt2, whereby PHLPP2 targets Akt1 for dephosphorylation [140].

## USING THE MOUSE TO GUIDE EFFECTIVE THERAPY

These data highlight a series of questions that can be resolved through the study of mouse models of PI3K-pathway activated breast cancer. Furthermore, the answers to these questions can help guide development of effective therapy. First of all, breast cancer patients with helical and kinase domain mutants show dramatically different survival [105]. Why is this? Perhaps helical mutants activate pro-migratory Akt2 dependent signaling and metastasis, whereas kinase domain mutants activate Akt1? If so, how does this operate at the molecular level? Alternatively, helical or kinase domain mutants may activate a different set of PIP3 targets such as ILK or SGK3, an estrogen-regulated AGC family kinase that is required for survival of the ERα-positive breast cancer cell line MCF7 [141, 142]. These questions can be readily addressed with mouse models. Firstly, a mouse model of Pik3ca^E545K^-induced breast cancer would have to be generated, analyzed and compared to an isogenic model of Pik3ca^H1047R^-induced disease. As helical domain mutants are more frequent associated with infiltrating lobular carcinoma and patients with these mutants show relatively poor survival [105], it would be interesting to determine whether tumors in a mouse model of *Pik3ca^E545K^*-induced breast cancer are lobular and metastatic, in contrast to the essentially non-metastatic tumors typically observed in *Pik3ca^H1047R^*-model mice [119].

Helical and kinase domain mutants are dependent on distinct signaling inputs for full PI3K pathway activation [48]. Consequently, a different network of oncogenic mutations may cooperate with each. Perhaps, mutations that activate Ras signaling will be selected for in tumors with helical domain mutants of *PIK3CA*, whereas tyrosine kinase receptor genes will be activated or amplified in tumors with kinase domain mutants. By using retroviral- or transposon-based insertional mutagenesis screens in *Pik3ca^(E545K vs H1047R)^*-model mice, or deep sequencing of *Pik3ca^(E545K vs H1047R)^*-model tumors, the cooperative network associated with each *Pik3ca* mutant can be defined and new combination therapies developed for patients with analogous *PIK3CA* mutant breast tumors. To test whether helical and kinase mutant alleles activate different PIP3 targets, such as Akt1, Akt2, ILK, SGK3 or guanine nucleotide exchange factors for Rho family proteins including Rac, signaling experiments can be performed on mouse tumors induced by each mutant. These experiments are particularly important as activated *Akt1* does not induce tumors in mice but overexpression of ILK does [143], and ILK has been shown to play a critical role in MMTV-Neu mouse mammary tumors [144]. Thus, with the genomic tools available today, sophisticated mouse models can be used to define how PI3K pathway genes cooperate with other mutations to control tumor dissemination.

Another unresolved question is the relationship between specific PI3K pathway mutations and breast tumor subtype. As noted above, *PIK3CA* mutations occur in many breast cancer subtypes but are rarely, if ever, seen in claudin-low tumors [99]. In contrast, *PTEN* mutations tend to cluster in triple negative tumors, a designation including basal-like and claudin-low breast cancers [107, 108, 145]. Potentially, this could be due to a specific biological response to each mutation type in the same *cell-of-origin*, and/or to distinct sensitivity of mammary stem cells or luminal progenitors to transformation by *PIK3CA* gain-of-function mutation versus *PTEN* deletion [146]. With respect to different biological response, activation of the PI3K signaling pathway at the level of HER2/Neu or IGF1 will result in activation of PI3K signaling together with activation of other signaling pathways stimulated by these receptors. This would not occur when the pathway is activated through *PIK3CA* mutation or *PTEN* deletion. Similarly, stimulation of the pathway by *PIK3CA* mutation will necessarily have different consequences than stimulation through *PTEN* inactivation, since PTEN protein also functions to inhibit the Src tyrosine kinase [35, 147, 148] and activate the APC-CDH1 complex [36, 37]. Once again, this question can be addressed using existing mouse models of breast cancer. Recently described *Pik3ca^H1047R^* breast cancer models are Cre-dependent [119, 121]. Therefore, by using multiple mammary specific Cre driver lines (eg. Wap-Cre versus MMTV-Cre [121] or K14-Cre) it will be possible to compare tumors that arise through expression of a *Pik3ca* mutant or deletion of *Pten* within the same mammary stem or progenitor cell.

Mouse models of breast cancer have been used to define signaling proteins and pathways that are required to initiate tumor formation, to sustain tumors and/or to promote metastasis. Indeed, as discussed above, this approach has shown that Akt1 and Akt2 perform very different roles with respect to growth and dissemination of HER2/Neu subtype tumors [134]. This approach can also be used to define the role or function of Akt1 and 2 downstream of mutant *Pik3ca* or *Pten* deletion in mice. The specific PI3K pathway involved in transformation may be different in tumors with amplified *HER2/Neu*, amplified *InsR/Igf-1R*, with mutant *PIK3CA*, with *PTEN* inactivation or with *Akt1^E17K^*. With the exception of activated *Akt1*, there are now mouse models for each of these, and with loxP/Cre-mediated deletion or even gene knockdown, the role of other components on the PI3K pathway can be determined. For example, the p110β subunit of PI3K may play an important role in *PTEN* inactivated tumors [149-152].

New pathways that activate PI3K signaling have been discovered. For example, the non-canonical IκB Kinase, IKKϵ, can phosphorylate Akt in a PI3K-dependent, but mTOR-independent, manner [153]. IKKϵ is overexpressed in most breast cancers and in 30% of cases this is associated with amplification of sequences on the long arm of human chromosome 1, including the IKKϵ gene, *IKBKE* [154]. As RTK/*PIK3CA*/*AKT1*/*PTEN* and IKK/NFκB are the two most frequently mutated pathways in breast cancer, any crosstalk between them may represent a critical therapeutic target [53]. Once again, a mouse model would help to probe this issue. Would a mouse model of *Ikbke*-induced breast cancer show cooperation with mouse models of activated *Pik3ca*? Indeed, *IKBKE* is amplified and overexpressed together with *PIK3CA* mutation in some human breast tumors suggesting that these mutations can cooperate (e.g. MCF7 cells show IKBKE amplification/overexpression and *PIK3CA^E545K^* mutation [141, 154]). By generating a mouse model of *IKBKE* overexpressing and *PIK3CA^E545K^* mutant breast cancer, it would be possible to define which PI3K pathway components are involved in growth and invasion in this context, and whether the specific oncogenic pathway is different from tumors with *Pik3ca^E545K^* and other cooperating mutations.

Finally, with the development of new mouse models to mimic specific forms of human breast cancer, it should be possible to perform high-throughput screens for chemicals or shRNAs that target mouse mammary tumor initiating cells while sparing normal mammary stem cells as well as other normal cells and tissues throughout the body. Thus, models that mimic each breast cancer subtype, with specific molecular lesions or combinations of lesions, can be developed and used to identify targets for combination therapy that will justify clinical trials on patients with analogous breast tumors. As activation of the mTOR pathway occurs in most breast tumors, it may also be possible to exploit this feature [155]. For example, in a mouse model of basal breast cancer with activated Ras, Igf1r signaling is required for survival [156]. Also, while direct mutational activation of the PI3K pathway in many breast tumors will preclude therapy based solely on caloric restriction [157], a number of reports have identified metabolic sensitivities associated with activation of specific oncogenic signaling pathways. For example, melanomas with activation of the Ras/Mapk pathway undergo apoptosis in response to leucine depletion. This effect is related to maintenance of activated TORC1 at lysosomes where it blocks autophagy, even in the absence of leucine [158]. As most breast tumors have sustained mutations that affect the very pathway used to sense nutrient availability, it is plausible that mouse models could prove useful in designing and testing nutrition based therapies.
